# Knowledge graph construction based on granulosa cells transcriptome from polycystic ovary syndrome with normoandrogen and hyperandrogen

**DOI:** 10.1186/s13048-024-01361-z

**Published:** 2024-02-12

**Authors:** Wensu Liu, Tianyu Tang, Jianwei Feng, Chunyu Wang, Lin Lin, Shengli Wang, Kai Zeng, Renlong Zou, Zeyu Yang, Yue Zhao

**Affiliations:** 1 Department of Cell Biology, Key Laboratory of Cell Biology, Ministry of Public Health, and Key Laboratory of Medical Cell Biology, Ministry of Education, School of Life Sciences, China Medical University, No.77 Puhe Road, Shenyang North New Area, Shenyang, 110122 Liaoning Province China; 2 Department of Ultrasound, Shengjing Hospital of China Medical University, Shenyang, 110004 Liaoning Province China; 3 Health Sciences Institute, China Medical University, Shenyang, 110122 Liaoning Province China

**Keywords:** Polycystic ovary syndrome, Hyperandrogenic polycystic ovary syndrome, Normoandrogenic polycystic ovary syndrome, Knowledge graph, Drug-gene interaction

## Abstract

PCOS is a widespread disease that primarily caused in-pregnancy in pregnant-age women. Normoandrogen (NA) and Hyperandrogen (HA) PCOS are distinct subtypes of PCOS, while bio-markers and expression patterns for NA PCOS and HA PCOS have not been disclosed. We performed microarray analysis on granusola cells from NA PCOS, HA PCOS and normal tissue from 12 individuals. Afterwards, microarray data were processed and specific genes for NA PCOS and HA PCOS were identified. Further functional analysis selected IL6R and CD274 as new NA PCOS functional markers, and meanwhile selected CASR as new HA PCOS functional marker. IL6R, CD274 and CASR were afterwards experimentally validated on mRNA and protein level. Subsequent causal relationship analysis based on Apriori Rules Algorithm and co-occurrence methods identified classification markers for NA PCOS and HA PCOS. According to classification markers, downloaded transcriptome datasets were merged with our microarray data. Based on merged data, causal knowledge graph was constructed for NA PCOS or HA PCOS and female infertility on NA PCOS and HA PCOS. Gene-drug interaction analysis was then performed and drugs for HA PCOS and NA PCOS were predicted. Our work was among the first to indicate the NA PCOS and HA PCOS functional and classification markers and using markers to construct knowledge graphs and afterwards predict drugs for NA PCOS and HA PCOS based on transcriptome data. Thus, our study possessed biological and clinical value on further understanding the inner mechanism on the difference between NA PCOS and HA PCOS.

**Supplementary Information:**

The online version contains supplementary material available at 10.1186/s13048-024-01361-z.

## Introduction

Polycystic ovary syndrome (PCOS) is a widespread heterogeneous endocrine and metabolic disease affecting 7%-12% reproductive-age women globally affected by the applied criteria [1, 2]. As defined by the well accepted diagnostic criteria Rotterdam Standard, patients with two of the three syndrome including hyperandrogenism, ovulatory dysfunction, and polycystic ovarian morphologic features on ultrasound were considered as PCOS [3]. However, it was also proposed that on the condition with the presence of both oligo- or anovulation and hyperandrogenism, ultrasound is not necessity for diagnosis. For adolescents, both oligo-anovulation and hyperandrogenism are required, while ultrasound was not recommended for diagnosis [4]. PCOS patients were normally suffered from cases such as obesity, dyslipidemia, insulin resistance, metabolic disorders, high risk of female infertility, hirsutism, cardiovascular disease and endometrial cancer [5]. However, since these traits were dramatically distinct and involved in several differentiated clinical phenotype, the research on etiology of PCOS is still insufficient.

Hyperandrogenemia is the core symptom of PCOS [6] and abundant evidences prove that excessive androgen may be the critical factor in the pathogenesis of PCOS. The animal model of PCOS also confirmed that high androgen level caused polycystic ovarian morphologic features [7, 8]. However, 18% to 25% PCOS patients were not caused by hyperandrogenemia but with other pathology including physiological and biochemical manifestion [9, 10], meaning still another subtype of patients existed with normal androgen levels. The PCOS subtype for these patients was as normoandrogen PCOS (NA PCOS), and relatively the PCOS subtype for the patients was hyperandrogen PCOS (HA PCOS)*.* NA PCOS patients express syndrome of ovulatory dysfunction accompanied with polycystic ovarian morphologic features and meanwhile HA PCOS patients express syndrome of hyperandrogenism, ovulatory dysfunction and polycystic ovarian morphologic features [3]. The significance for dividing PCOS into HA PCOS and NA PCOS was to separate two groups of distinctive patients with different pathology, syndromes and adverse metabolic profile through life [11]. As syndromes of two subtypes of PCOS were greatly differentiated, it has been proposed the two subtypes might actually be different types of diseases [3, 9]. However, the research on the mechanism on the difference of HA PCOS and NA PCOS was rare and unreliable.

In order to distinguish the difference between NA and HA PCOS, it was necessary to ensure the most essential cell subtype for ovary. Ovary is composed of four major cell subtypes: theca cells, granulosa cells, cumulus cells and stromal cells. Androgen acts via androgen receptor (AR) in ovary to promote follicle growth and steroidogenesis. AR is expressed in oocytes, granulosa cells, and theca cells. AR is temporally regulated during follicular development. In former studies, AR was specifically knocked out in all four major ovarian cell subtypes, and the results disclosed that androgen played distinct roles in each cell subtype. Mouse knockout studies also showed that AR was expressed most critically in granulosa cells within the four cell subtypes for normal follicular development and subsequent ovulation [12–14] and thus might be significantly correlated to PCOS. Thus study on transcriptome of granulosa cells might be essential to discover the traits on NA PCOS and HA PCOS and further understand the pathogenesis of the two subtypes of PCOS.

Omics and multi-omics studies have been performed on PCOS transcriptome data to investigate the pathogenesis for PCOS and search PCOS biomarkers [15–17]. While however, the microarray or RNA-seq analysis on NA PCOS and HA PCOS were rare in NCBI Gene Expression Omnibus (GEO) datasets or published papers, and comparative transcriptome study on NA PCOS and HA PCOS was not yet published. To fill-in this vacancy, we performed microarray analysis on granulosa cells from NA and HA PCOS patients and normal individuals (4 female subjects for each group) to further identify potential marker genes for NA PCOS and HA PCOS subtypes. Sample numbers were comparably limited due to the relative difficulty on acquiring PCOS samples. The microarray data were afterwards processed to identify differentiated and afterwards specific genes for NA PCOS and HA PCOS. Further functional analysis filtered the specific genes into list of potential marker genes for NA PCOS and HA PCOS. Deeper analysis finally identified NA PCOS marker IL6R and CD274, and meanwhile HA PCOS marker CASR. Subsequent RT-qPCR and Western blotting experiments validated differential expression of these markers on NA PCOS and HA PCOS. Afterwards, we applied combined methods of Apriori Rules Algorithm [18, 19] and co-occurrence analysis on downloaded PubMed PCOS abstracts and drug-gene interaction analysis to construct causal knowledge graph for NA PCOS and HA PCOS markers and female infertility. For our last step, drug-gene interaction analysis was performed to predict drugs for HA PCOS and NA PCOS.

## Material and method

### Patient selection and ethical statement

**Fig. 1:**
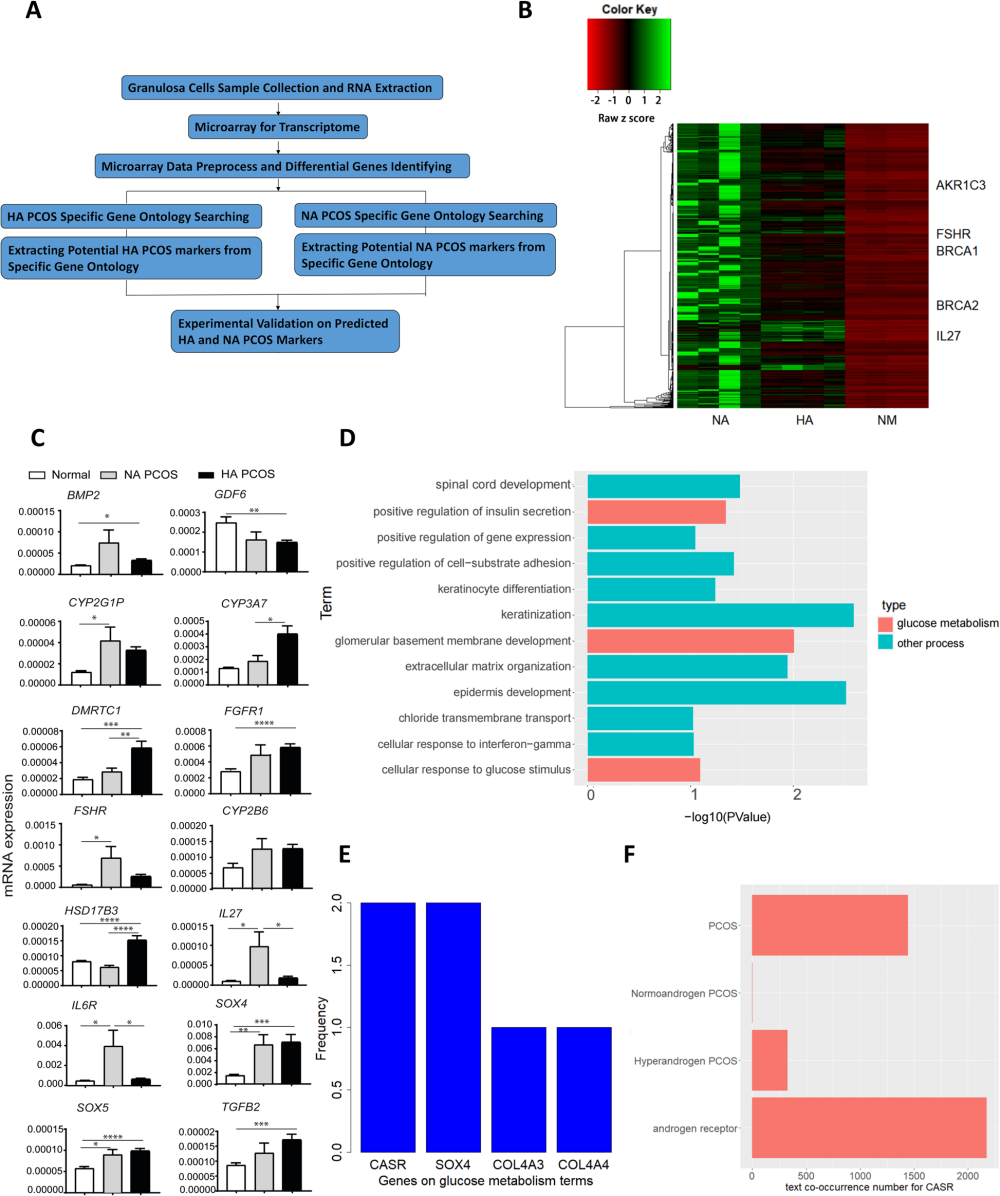
PCOS Data Acquisition and Identifying potential markers for HA PCOS specific genes. **A** Flowchart of the process of data procession progress, experimental design and analysis procedure for the microarray data. **B** Heatmap illustrating the 615 HA PCOS, NA PCOS and NM differentiated genes in GEO datasets and the microarray dataset with PCOS and some known PCOS and ovarian or breast tumor markers labeled on the right side. **C** The mRNA level measured by quantitative real-time PCR on the top 14 expressed genes on NA PCOS, HA PCOS and Normal (NM) groups for our microarray data. Data are expressed as means ± SEM. *p ≤ 0.05, Student t test. **D** Barplot showing the DAVID biological process analysis terms within the HA PCOS specific genes. Glucose metabolism terms and other terms were labeled red, and other terms were labeled as blue. **E** Barplot illustrating the HA PCOS specific genes on glucose metabolism terms(x-axis) and the corresponding number of glucose metabolism terms which each gene belonged (y-axis). **F** Co-occurrence results for CASR with PCOS, NA PCOS, HA PCOS and androgen receptor respectfully

All study participants were women at the Center of Reproductive Medicine, Shengjing Hospital of China Medical University (Shenyang, China). A diagnosis of PCOS was based on the Rotterdam criteria, which was that patients with two of the three syndrome including hyperandrogenism, ovulatory dysfunction, and polycystic ovarian morphologic features on ultrasound were considered as PCOS. We enrolled 12 women in this study (Fig. 1e), 8 women with PCOS (PCOS group) and 4 body mass index- and age-matched healthy women (control group). The subjects’ characteristics are provided in Table S1. This is a prospective study. A total of 20 samples were screened during the two-month period and the exclusion criteria were as follows: (1) diagnosed with other endocrine system diseases, such as diabetes. (2) complicated with Severe primary diseases associated with cardiovascular system, liver and kidney, tumor, infection and other autoimmune diseases. (3) lack of medical history information and imperfect laboratory indexes. Among the 8 women with PCOS, we separated the patients into HA PCOS group and NA PCOS group according to the following citeria: (1) Clinical and/or biochemical signs of hyperandrogenism; (2) Oligo- and/or anovulation; (3) Polycystic ovaries. In accordance with two or more of the above three items, polycystic ovary syndrome can be diagnosed after excluding other etiologies (congenital adrenal hyperplasia,androgen-secreting tumors, Cushing’s syndrome). Clinical manifestations and/or diagnostic criteria of hyperandrogenemia: multiple acne in forehead, cheek, nose and mandible for more than 3 months, Ferryman-Gallwey score ≥ 7, pseudo-acanthosis nigricans, moderate to severe seborrheic dermatitis, androgen alopecia (sparse hair from top to anterior hairline) and/or testosterone higher than 0.55 ng/ml (laboratory standard of Shengjing Hospital). All patients were with age between 22–35 years since birth. The study protocol was approved by the Research Ethics Committee of China Medical University. All participants provided signed informed consent.

### Ovarian stimulation, granulosa cell collection and RNA extraction

On the second day of the menstrual cycle, the number of follicles in bilateral ovarian sinuses and the condition of endometrium were monitored by transvaginal ultrasound, combined with serum hormone levels (FSH, LH, estradiol, progesterone, testosterone, prolactin). To promote follicular growth, control and PCOS patient ovaries were stimulated with recombinant follicle-stimulating hormone (FSH) (Merck-Serono, Switzerland) after pretreatment with gonadotropin-releasing hormone agonist (Diphereline, Ipsen Pharma Biotech, France). FSH stimulation was started when down-regulation was confirmed by vaginal ultrasonography and by measurements of serum estradiol and luteinizing hormone. Ultrasonography was used to evaluate follicular development from day 5 of stimulation until the day of follicular maturation. When at least 1 follicle grew to 18 mm in diameter, 250 μg of recombinant hCG (Ovidrel, Merck-Serono, Switzerland) was administered, and oocyte retrieval was performed 34–36 h later. Follicle size was determined based upon the average follicular diameter, and follicular fluid samples from mature follicles (17–22 mm) and matched-immature follicles (8–13 mm) were stored separately. The granulosa cell samples were centrifuged at 12,000 g for 10 min. The precipitate is a mixture of GCs, red blood cells and white blood cells. The red blood cell lysate of 3 times volume was added to the precipitate and centrifuged 5 min at 37 ℃ for 5 min at 1500 rpm. The supernatant was discarded and washed twice with PBS to Collect granulosa cells. Then RNA was extracted from granulosa cells. The quality and integrity of RNA samples were analyzed using the Agilent 2100 Bioanalyzer (Agilent Technologies, Inc., Palo Alto, CA, USA) [20].

### Microarray data analysis

Each RNA sample was generated by Agilent SurePrint G3 Human GE 8 × 60 K Microarray kit (Agilent Technologies Canada Inc., Mississauga, ON) with standardized protocol as described [21]. Microarray data were applied with background adjustment and processed applying Feature Extraction software (version 10.7.1.1) and analyzed using Genespring software (version 13.1) in matching reference genome Hg19 and tpm calculation. Genes with mean tpm < 0.1 were removed. Normalization, RMA summarization and Student’s t test were performed using R limma package (version 3.34.9). Student’s t test was calculated based on the expression value of the HA PCOS group, NA PCOS group and normal tissue sample group and then applied to identify HA PCOS and NA PCOS differentiated genes. Genes with Students’ test *p* value < 0.05 and fold change > 2 or < 0.5 for either 2 groups were regarded as differentiated genes. Genes both differentiated for NA PCOS vs normal tissue and NA PCOS vs HA PCOS were regarded as NA specific genes. Genes both differentiated for HA PCOS vs normal tissue and HA PCOS vs NA PCOS were regarded as HA specific genes.

### Further analysis for microarray data

Pie plot was generated using R package plotrix [22] (version 3.7.4). Barplots were plotted by R ggplot2 package [23] (version 3.1.0). Venn analysis was visualized by R package VennDiagram [24] (version 1.6.20). Heatmaps were generated using R gplots package [25] (version 3.0.1.1). Gene ontology (GO) analysis was performed according to DAVID database and GO figure was generated using R package ggplot2 (version 3.1.0). Colors for all figures were generated by R RColorBrewer package [26] (version 1.1.2).

### Co-occurrence analysis using PubMed

Co-occurrence counts were calculated by R RISmed package [27] (version 2.1.7) and self-written R code. The principle was inputting the searching word in the format “Term A and Term B” into PubMed and the co-occurrence count was the number of articles displayed by PubMed. Co-occurrence count was reflecting the strength of relationship between term A and term B [28]. Term A and Term B might be genes or diseases or Medical Subject Headings (MeSH) terms.

### Protein–protein interaction network analysis

Protein–protein interaction network was calculated by STRING online database using human version of 9606.protein.links.v11.0 and the “Multi-protein” function [29, 30]. “ + ” button was pressed once on the situation on which the inputted proteins were not linked. “Experiment” and “Databases” filtering was used on the situation on which the links between inputted proteins were substantially high.

### Deeper gene search based on NA PCOS gene ontology analysis

For the analyis in this part, the BP and KEGG terms with *p* value < 0.05 were removed and arranged in ascending order on *p* value. BP or KEGG all terms meant all terms with *p* value < 0.05 and meanwhile the top 20 terms meant the 20 BP or KEGG terms with least *p* value. The Biological Process gene oncology analysis from DAVID on NA PCOS specific genes was firstly applied [31]. The immunity terms and the terms correlated with immunity were manually picked and the genes on these terms with appearing frequency > 5 were regarded as potential marker genes since number of terms was high. This process was afterwards repeated on the top 20 GO terms, all KEGG pathway terms and top 20 KEGG pathway terms. The genes satisfying the four conditions were regarded as potential marker genes. STRING ppi analysis (using Experimental and Database filtering) was applied on these potential marker genes to remove genes with no connection with other genes. Further filtering was applied by co-occurrence analysis with the inputting format “potential marker gene name and PCOS” and the marker gene with co-occurrence number > 0 was reserved. Finally, the selected GO terms were applied with co-occurrence analysis using the inputting term “GO term and PCOS”. The GO term with the most co-occurrence number was reserved. The genes on this GO term were regarded as NA PCOS functional marker.

### Deeper gene search based on HA PCOS gene ontology analysis

The Gene Ontology Biological Process analysis from DAVID on HA PCOS specific genes was firstly applied. The BPs with *p* value < 0.05 were removed. The terms highly correlated with glucose were manually picked and the genes on these terms with appearing frequency > 1 were regarded as potential marker genes. Further filtering was applied by co-occurrence analysis using the inputting format “potential marker gene name and PCOS”. The gene with co-occurrence number > 0 was reserved as HA PCOS functional marker.

### Classification markers identification and data integration

Each gene was classified into HA PCOS, NA PCOS or normal (NM) group based on the group with the highest expression for the gene. 10–50 genes were randomly selected for each group. And afterwards for each sample, all genes were arranged from highest to lowest and the ranking for each gene was recorded. The predicted category of sample was decided by the category with the highest mean ranking of group of genes. This process was repeated for 50 million times. The predicted category of the samples should be the same with the actual category of the samples. We completed the calculation and reserved all set of genes that satisfied this criteria. Then our microarray data was merged with downloaded gene omnibus (GEO) transciptome datasets on PCOS and then normalized*,* and used each set of genes to classify the samples by the category with the highest mean ranking of group of genes. The matching rate was calculated using the following equation: matching rate = number of samples with accurate predicted categories/all number of samples. The groupings with the highest matching ratio for downloaded datasets (the sample predicted in HA PCOS and NA PCOS category comparing the actual PCOS category, and the sample predicted in NM category comparing the actual NM category) were reserved. The HA PCOS, NA PCOS and NM genes on the best reserved grouping were regarded as classification markers. Finally, the selected set of genes were regarded as classification markers and used to classify the downloaded datasets into HA PCOS, NA PCOS and NM groups. The predicted category of sample on the downloaded GEO datasets was decided by the category with the highest mean ranking of group of classification markers. And thus the downloaded datasets were capable of merging with our microarray data on integrating samples by the same genes.

### Apriori rule analysis on merged transcriptome data

The merged transcriptome expression tables were independently discretized based on the expression level ranking for each gene on different samples. The samples possessing less than two genes with expression > 0 were removed. On total, the discretization was performed for a 4-element quantile discretization (separated by 75%, 50% and 25% rank) and a 2-element quantile discretization (separated by 50% rank). Minimum discretization value was defined as the value less than 25% rank for 4-element discretization and 50% rank for 2-element discretization. Association rules calculation was performed independently for each discretization method. Each gene pair (gene A and gene B) from the gene co-expression filtering results was nominated as a rule and afterwards the confidence and support for each rule were calculated using the following equations:

$$\mathrm{confidence\ A}\to {\text{B}}=\frac{{\text{n}}({\text{A}}\ =\ \mathrm{B }\ \cap\ {\text{A}}\ >\ {\text{min}}({\text{A}})\ \cap \ {\text{B}}\ >\ {\text{min}}({\text{B}}))}{{\text{n}}({\text{A}}\ >\ {\text{min}}({\text{A}}))}$$

$$\mathrm{support\ A}\to {\text{B}}={\text{n}}({\text{A}}>{\text{min}}({\text{A}}))+{\text{n}}({\text{B}}>{\text{min}}({\text{B}}))$$

n() means the number of the subject, for instance, n(A > min(A)) means number of A whose value is above minimum value of A, and also n(A = B) means number of paired A and B which satisfies A value equaling B value. $\cap$ stands for intersection for the left object and the right object.

Afterwards, confidence and support for the two different discretization methods were summed. Rules with summed confidence > 0.6 and summed support > 30 were reserved. The direction (arrow) showed potential causal relationship pointing from the potential driver gene (upstream, or cause) to the potential driven gene (downstream, or result). The method has been uploaded to GitHub as WLAR method (https://github.com/dawnplague/WLAR.git).

### PubMed abstracts download and preprocessing

All available PubMed abstracts from 2010.8.1–2022.8.1 were downloaded and processed by R pubMR package [32] (version 0.0.0.2). The abstracts containing HA PCOS markers and HA PCOS differentiated genes were deemed as HA PCOS abstracts. The abstracts containing NA PCOS markers and NA PCOS differentiated genes were deemed as NA PCOS abstracts. The MeSH words with the sub major words containing “metabolism”, “chemical”, “genetics” etc. were extracted for each abstract. All possible combination of paired PubMed ID (PMID) and MeSH term/marker genes were recorded for the next step.

### Apriori rules algorithm analysis and knowledge graph construction

The association rules were calculated on the paired PMID and MeSH term/marker gene table from the previous step. This step was respectively performed on NA PCOS and HA PCOS. Each two MeSH term/marker gene (A and B) were forming a pair and the confidence of the rule was calculated as the division of number of intersecting PMIDs for A and B by number of the PMIDs of the A. The support of the rule was calculated by adding number of PMIDs of A and B.The result showed the causal relationship pointing from A to B. This part was using the same equations as the previous described association rules equations for transcriptome data. The causal relationship for B pointing to A was also calculated. The rules with confidence > 0.1 and support > 0.001 were reserved. Afterwards, the Apriori Rules Method was applied on the calculated rules to form rules with 3 elements. The confidence of rules with more elements were selected by multiplying all confidences of the corresponding 2-element rules and then the 3-element rules with confidence < 0.1 were removed. The rules with the 3^rd^ element as “female infertility” were reserved. The reserved 3 rules were splitted into 2-element rules, and then combined with the association rules results for the existing genes. The causal knowledge graph was respectively built for NA PCOS rules and HA PCOS rules. The causal network visualization was realized by Cytoscape [33] (Version 3.9.1).

### Kaplan–Meier’s survival analysis

Kaplan–Meier’s Survival analysis was performed by GEPIA 2021 [34, 35]. The survival *p*-value for GENE + between GENE- for each tumor was extracted. Hazard ratio value for each gene on each tumor was extracted.

### Drug-gene interaction analysis

Drug-Gene interaction data were downloaded from Gene Regulatory Network database (GRNdb) [36]. Drugs interacted with HA PCOS functional/classifcation markers were regarded as drugs for HA PCOS. Drugs interacted with NA PCOS functional/classifcation markers were regarded as drugs for NA PCOS. Drugs both regarded as HA PCOS and NA PCOS drugs were removed from the 2 groups. The reserved drug-gene interaction was then visualized by Cytoscape (version 3.9.1).

### Quantitative real-time PCR

Total RNAs were isolated from cells using the TRIzol reagent(TAKARA). RNAs were reversely transcribed into cDNA with PrimeScript RT-qPCR kit (TAKARA) in a 20 ml reaction. qPCR assays were performed with SYBR premerase Taq kit (TAKARA) on LightCycler96 (Roche) and the sequences of primer pairs used in the present study are shown in Table S2.

### Western blotting and antibodies

For potential gene (IL6R and CD274), total protein was extracted from an additional three non-PCOS granulosa cells and three PCOS granulosa cells, and 20 μg of protein per lane was loaded and separated by sodium dodecyl sulfate–polyacrylamide gel electrophoresis. Proteins were then transferred to polyvinylidene fluoride (PVDF) membranes and blocked with 5% nonfat milk in Tris-buffered saline with Tween-20 (TAKARA) for 1 h. The membranes were incubated overnight at 4 °C with one of the following primary antibodies: IL6R (p-ERK; diluted 1:500), CD274 (diluted 1:1000), and glyceraldehyde 3-phosphate dehydrogenase (GAPDH; diluted 1:5000) (Santa Cruz Biotechnology, Inc., Santa Cruz, CA, USA). Next, PVDF membranes were incubated for 60 min at room temperature with secondary antibodies (Santa Cruz Biotechnology, Inc.), and immune complexes were detected by chem-iluminescence using a Western blotting analysis system (TAKARA). Relative protein levels per sample were then normalized to GAPDH signals.

### Statistical analysis

Clinical features of interest were selected as dependent variables. The Mann–Whitney U test, Student’s t-test and paired t-test of variance were used where appropriate for statistical analyses. Data from at least three independent measurements were presented as means ± standard deviation of the mean (SEM). The SYSTAT statistical graphics software package (SYSTAT Software, Inc., San Jose, CA, USA) was used for analysis, and a probability (*P*) value of < 0.05 was considered statistically significant. *p < 0.05, **p < 0.01, ***p < *0.001.*

## Results

### Differential analysis on the granulosa cells microarray data in identifying HA and NA PCOS specific genes

The combined flowchart for first part of our study was illustrated (Fig. 1a)*,* and the main intention was to identify functional and classification markers for HA and NA PCOS and then construct knowledge graph. Since the studies on PCOS were insufficient, we used the concept of co-occurrence from philology to evaluate the relationship between 2 concepts. Co-occurrence analysis was using the number of papers which simultaneously included the 2 concepts to reflect the relationship between the 2 concepts. Higher number of co-occurrence meant stronger relationship. The relationship disclosed by co-occurrence analysis was a reflection of predicted relationship based on current and published studies. Co-occurrence analysis based on the abstract of PubMed published papers was performed to stat the correlation between PCOS and four subtypes of ovarian cells including granulosa cells, cumulus cells, theca cells and stromal cells. Granulosa cells were with the highest co-occurrence counts within the four cell subtypes with PCOS (Fig. S2a). Microarray analysis was then performed on granulosa cells from 12 individuals. Clinic information illustrated these individuals were within three groups: normal individuals, HA PCOS individuals and NA PCOS individuals (Table S1). Groups were separated based on clinical symptoms and testosterone level, and afterwards granulosa cells in all groups were collected and assayed. Pearson’s correlation analysis for all genes based on the expression value of the 12 samples showed high quality for our microarray data (Fig. S2b). We further validated the precision of the microarray result through checking if the expression pattern of the 14 PCOS top-expressive genes in microarray data was concordant with RT-qPCR results on the same 14 genes and the results of RT-qPCR showed similar pattern (Fig. 1c). Box Whisker plot, matrix plot, principal component analysis (PCA) and sample clustering analysis further confirmed the quality of our microarray data (Fig. S1a-d).

The microarray data was then preprocessed and normalized. Differential analysis was afterwards performed and showed 615 HA PCOS, NA PCOS or NM differentiated genes including a series of known PCOS and feminine tumor markers (Fig. 1b). The existence of these markers in differentiated genes confirmed the clinical research value for our microarray data. We further filtered the genes expressed highest in HA PCOS or NA PCOS, and with statistical difference with the other 2 groups, and these genes were named HA or NA PCOS specific genes. On this filtering, 130 NA PCOS specific genes and 43 HA PCOS specific genes were identified. Functional analysis was afterwards further applied on the selected specific genes to deeper filter NA PCOS and HA PCOS potential marker genes.

### Functional analysis on HA PCOS specific genes

Functional marker genes were markers genes with biological functions and thus were important in understanding the inner mechanism of PCOS. The main idea for identifying functional markers was to find the best genes for further analysis and experimental validation from large number of candidate genes. DAVID Gene Ontology (GO) analysis was performed on the 43 HA PCOS specific genes (Fig. 1d) [31]. From the GO results, we noticed high percentage of the terms were the terms associated with glucose metabolism, and this fact was explained by the close interaction between PCOS and insulin. The glucose metabolism terms possessed approximately 25% of all terms (Fig. S2c). The DAVID KEGG Pathway (KEGG) results for HA PCOS were not showing patterns for our interest. For this reason, HA PCOS functional markers should also be the genes on terms associated with glucose metabolism. Thus for the next step, genes on these glucose metabolism biological process (BP) terms were extracted, and the number of terms containing these genes were counted (Fig. 1e). CASR and SOX4 were the only genes appearing on more than 1 glucose metabolism term and then selected. STRING ppi analysis were afterwards performed on CASR and SOX4 using multi-protein function and pressing “ + ” once to display the inter-proteins between CASR and SOX4 (Fig. S2d). CASR possessed more ppi interactions than SOX4, implying CASR was more engaged in biological processes. Meanwhile, co-occurrence analysis showed that CASR was co-occurred with PCOS, HA PCOS, NA PCOS and androgen receptor, while SOX4 was merely co-occurred with androgen receptor (Fig. 1f, Fig. S2e). According to these results, SOX4 was consequentially removed, and CASR was selected as new HA PCOS functional marker.

### Functional analysis on NA PCOS specific genes

**Fig. 2:**
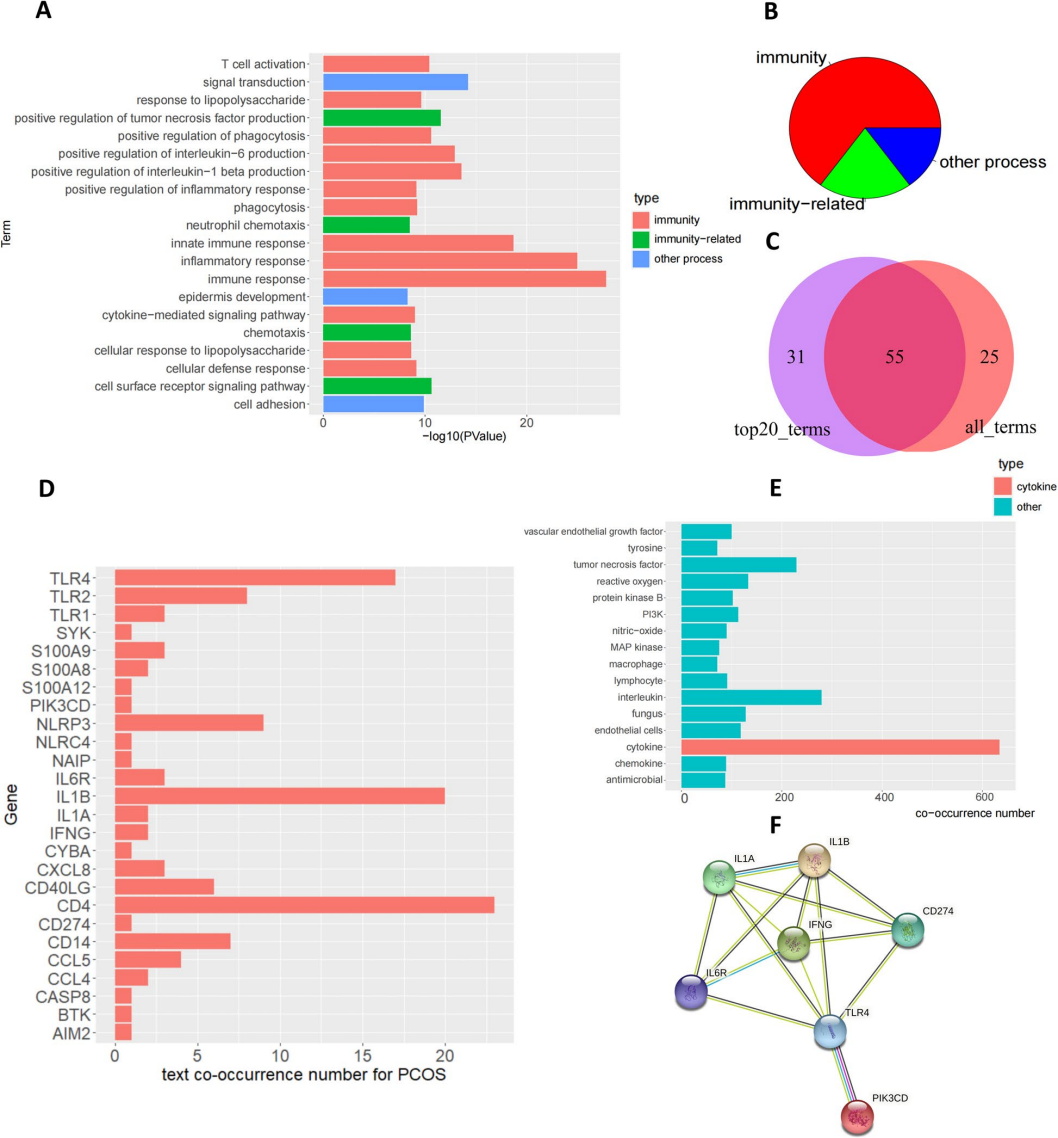
Applying Gene Ontology on specific genes to identify potential markers for NA PCOS. **A** Barplot showing the top 20 DAVID biological process analysis terms within the NA PCOS specific genes. Immunity terms and immunity related terms were labeled red and green, and other terms were labeled as blue. **B** Pie plot illustrating the percentage of immunity terms, immunity-related terms and other terms within the top 20 DAVID biological process analysis terms. **C** Venn plot showing number of NA PCOS specific genes on all immunity terms (> 5 frequency and with STRING connection) and top 20 immunity terms (> 2 frequency and with STRING connection). **D** Barplot illustrating the text co-occurrence number with PCOS for each candidate gene. **E** The GO terms with at least two candidate genes were listed (y-axis) and used to calculate text co-occurrence number with PCOS (x-axis). The most co-occurred term was labeled red. **F** The candidate genes within cytokine term were collected and performed with protein–protein interaction analysis by STRING analysis

DAVID GO analysis was performed on the 130 NA PCOS specific genes [31]. BP analysis results showed the majority terms within the top 20 terms were immunity terms or immunity related terms (Fig. 2a,b). Meanwhile, the majority terms within all BP terms were immunity or immunity related terms (Fig. S3a,b). The KEGG pathway analysis results showed the majority pathways within the top 20 pathways were immunity pathways or immunity related pathways (Fig. S3c,d). Meanwhile, the majority pathways within all KEGG pathways were immunity pathways or immunity related pathways (Fig. S3e,f). These results indicated the close relationship between NA PCOS and immunity. For this reason, NA PCOS functional markers should also be immunity or immunity related genes. We extracted the 100 genes which appeared on immunity or immunity related BP terms or pathways on all the following conditions: BP top 20 terms, BP all terms, KEGG top 20 terms and KEGG all terms.

For further filtering, appearance frequency of the potential marker genes on all immunity or immunity-related BP terms were counted (Fig. S4a). The 100 genes with more than 5 frequency were reserved. Subsequent STRING protein–protein interaction (ppi) analysis on the 100 genes using experimental and database links found 80 of the 100 genes were connected together (Fig. S4b). The same procedure was also performed on top 20 immunity or immunity-related BP terms, and the 86 genes out of 100 genes were with frequency > 2 and connected together (Fig. S4c,d). 55 intersected genes were within the 80 genes from all BP results and the 86 genes from top 20 BP results (Fig. 2c). And thus the 55 genes were regarded as potential marker genes. Further co-occurrence analysis with the word “PCOS” reserved 26 genes out of the 55 genes with co-occurrence number > 0 (Fig. 2d). Afterwards, we calculated the frequency of immunity or immunity-related GO BP terms for the 26 genes and selected the terms with at least 2 times and then performed co-occurrence analysis with the word “PCOS” on these terms. The term “cytokine” was mostly co-occurred (Fig. 2e). The 7 genes on the term “cytokine” out of the 26 genes were selected (Fig. 2f). Within all “cytokine” terms, the “response to cytokine” term was more related to immunity than the other terms. For this reason, the genes on this term, IL6R and CD274, were selected as new NA PCOS functional markers.

### Further validation for new NA PCOS and HA PCOS markers

Within new NA PCOS markers, IL6R (interleukin 6 receptor) belongs to a subunit of the interleukin 6 (IL6) receptor complex. As an IL6R ligand, IL6 is a potent pleiotropic cytokine that regulates cell growth and differentiation and plays important role in immune response.IL6R has been studied to be correlated with PCOS. The research found between PCOS patients and normal individuals, the most observed inflammation status of IL6R has been resulted from relative obesity or insulin resistance, and meanwhile not independent character of PCOS [37, 38]. The IL6R difference in NA and HA PCOS has not been reported. CD274 (CD274 molecule), also named PDL1, encodes an immune inhibitory receptor ligand which was involved in the immune escape process for tumor. The expression of CD274 in tumor cells is regarded as prognostic in many types of human malignancies such as colon cancer and ovarian cancer [39–41]. However, relationship of CD274 with PCOS has not yet been reported. Meanwhile, for new HA PCOS markers, CASR (calcium sensing receptor) is a plasma membrane G protein-coupled receptor that senses small changes in circulating calcium concentration. Previous studies have suggested that the pathological mechanism of insulin resistance in PCOS is related to calcium homeostasis, and CASR as an important calcium regulator may play an important role in PCOS pathogenesis [42–44]. Conclusively, these studies indicated that IL6R, CD274 and CASR might engage in important biological processes in NA PCOS or HA PCOS.

**Fig. 3:**
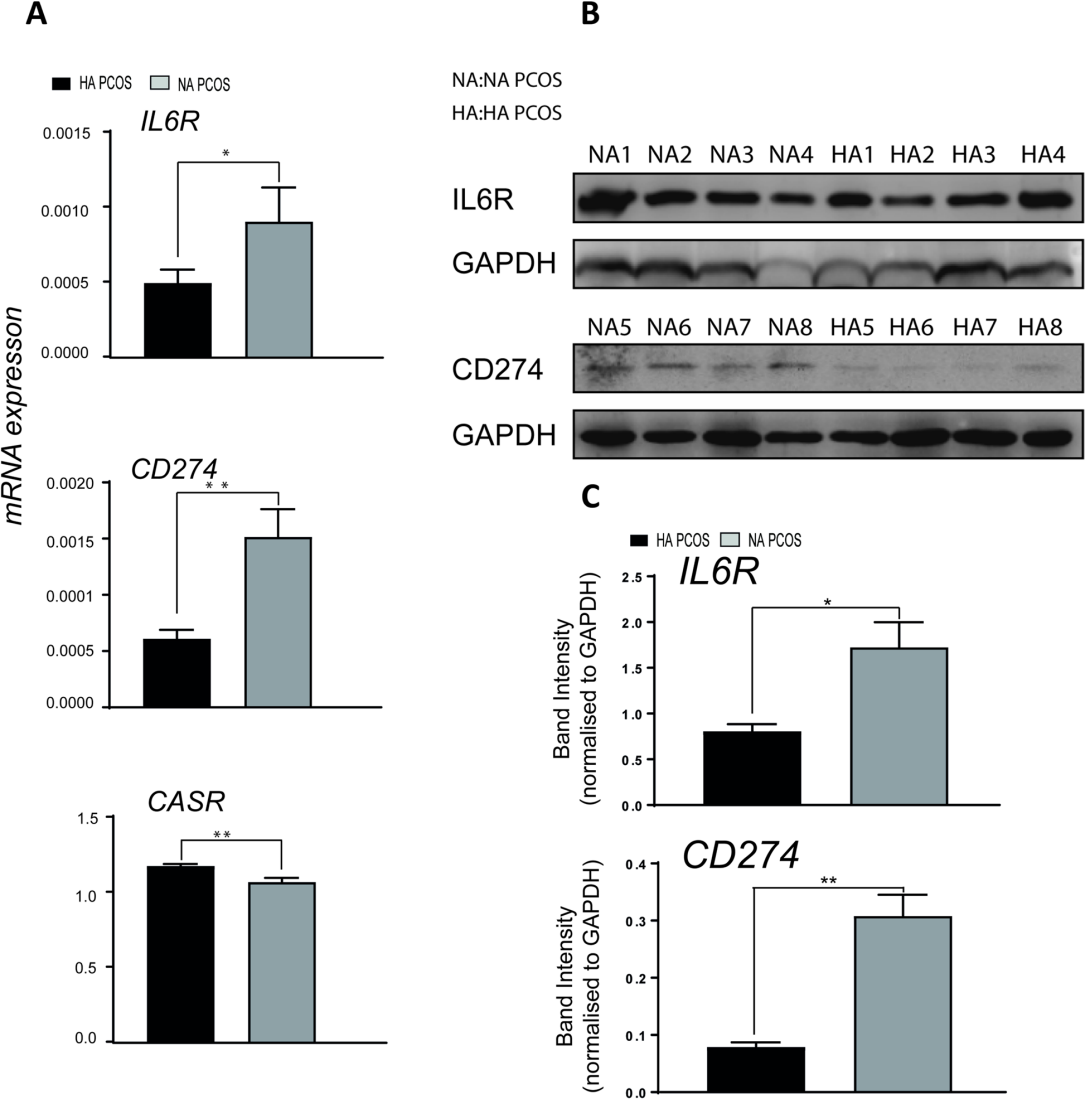
Experimental Validation of HA and NA PCOS Potential Marker in mRNA and protein level. **A** The mRNA expression of IL6R, CD274 and CASR in human granulosa cells from patients with HA PCOS (*n* = 4) and NA PCOS (*n* = 4) measured using quantitative real-time PCR. Data are expressed as means ± SEM. *p ≤ 0.05, Student t test. **B** and **C** The expression of IL6R and CD274 in human granulosa cells from HA PCOS (*n* = 4) and NA PCOS (*n* = 4) patients were subjected to Western blot analysis and values were normalized to GAPDH expression. Data are expressed as average and standard error of the mean (SE). The statistical analysis was performed using Student’s t test; *p ≤ 0.05 is considered significant

RT-qPCR was then applied to further confirm the expression of IL6R, CD274 and CASR in human granulosa cells. Groups were set as HA PCOS group and NA PCOS group. Notably, RT-qPCR showed that the expression level of IL6R and CD274 were significantly higher in NA PCOS than HA PCOS (Fig. 3a), and the result was in accordance with our former analysis that IL6R and CD274 were new NA PCOS markers. Meanwhile, RT-qPCR also illustrated that the expression level of CASR was higher in HA PCOS than NA PCOS (Fig. 3a), and it is also consistent with the result that CASR was new HA PCOS markers.

The protein level of NA PCOS specific genes IL6R and CD274 was further measured by Western blotting. Groups were set as HA PCOS group and NA PCOS group. Western blotting results further showed IL6R and CD274 were expressed on significantly higher protein level in NA PCOS than HA PCOS (Fig. 3b) and the similar results were also illustrated in level of band intensity (Fig. 3c). Combined with the experimental result of RT-qPCR and Western blotting, IL6R and CD274 were validated to be NA PCOS specific genes and new markers, and meanwhile CASR was validated to be HA PCOS specific genes and new marker.

### Identifying classification markers for HA PCOS and NA PCOS

**Fig. 4:**
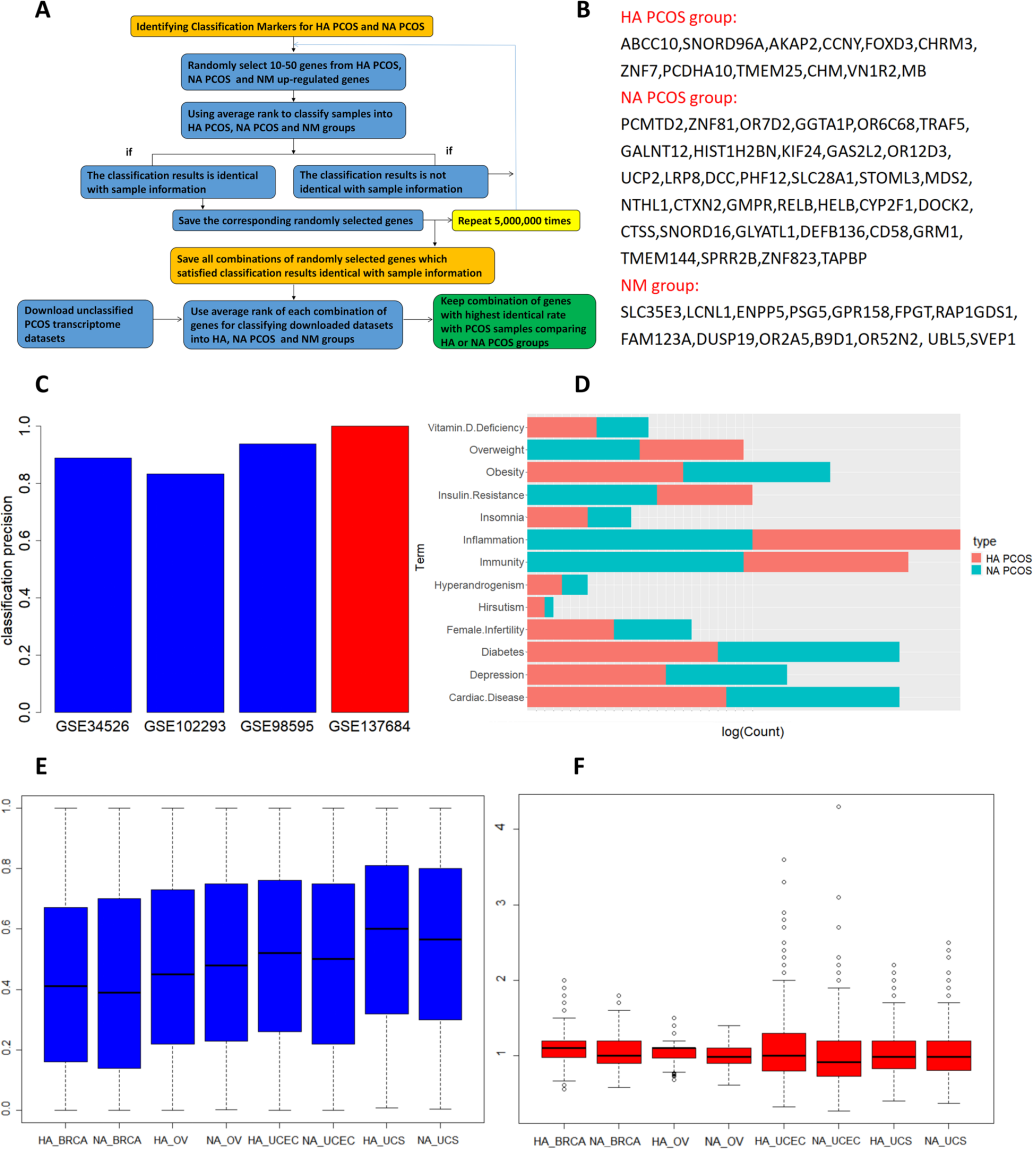
Identifying HA PCOS and NA PCOS Classification Markers and Using Classification Markers to Integrate with downloaded PCOS transcriptome data. **A** Flowchart for the process of identifying HA PCOS and NA PCOS Classification Markers. **B** The selected classification markers for HA PCOS, NA PCOS and Normal (NM) groups. **C** The classification precision for downloaded datasets of GSE34526, GSE102293 and GSE98595 (blue) and our data GSE137684 (red). **D** The logarithm of mean co-occurrence counts of HA PCOS and NA PCOS up-regulated genes after merging with metabolic anomaly correlated with PCOS. **E**, **F** The average *p* value (**E**) and hazard ratio (**F**) for Kaplan–Meier Suivival Anaysis on the 4 female specific cancers (BRCA = breast cancer, OV = ovarian cancer, UCEC = endometrial cancer, UCS = uterine cancer) for HA PCOS and NA PCOS up-regulated genes

Which markers were capable of classifying unassorted PCOS samples into HA and NA PCOS samples was of high clinical importance. We applied a random method on classification markers identification (refer to Methods, 9th section, Fig. 4a). Firstly, each gene was classified into HA PCOS, NA PCOS or normal (NM) group based on the group with the highest expression for the gene. Secondly, 50 million cycles of calculations were performed on the random set of 10–50 genes within each group. The predicted category of sample was decided by the category with the highest mean ranking of group of genes. The predicted category of the samples should be the same with the actual category of the samples. We completed the calculation and reserved all set of genes that satisfied this criteria. For instance, 30 NA PCOS genes were randomly picked, and the mean ranking of the 30 genes on one sample was 17,000 of the 20,000 genes, and was higher than HA PCOS (11,000) and NM (9,000) and thus this sample was classified as NA PCOS. If this sample was also NA PCOS from clinical information, and if the same was true for all samples, this random set of genes might be classification markers.

Five hundred seventy-eight sets of genes were capable of classifying the sample of our microarray data. If the classification markers were reasonable, these markers should also be applied for classifying PCOS sequencing data for published work of the other researchers. Subsequently, the 578 reserved sets of genes were further filtered using most highly confident downloaded transciptome data from GEO datasets GSE34526, GSE102293 and GSE98595. Though the 3 datasets were unclassified, we could still apply the known PCOS and NM groups for these datasets, with NA PCOS or HA PCOS comparing PCOS, and NM comparing NM. The set of genes with highest matching rate were selected and regarded as classification markers (Fig. 4b). The matching rate for the classification markers on our microarray data and downloaded datasets was also illustrated (Fig. 4c). Afterwards, the classification markers were applied on the downloaded datasets and classified the PCOS samples from downloaded GEO datasets into HA and NA PCOS samples. These HA and NA PCOS samples were merged into our microarray data and the heatmap of combined datasets were visualized (Fig. S5a). HA or NA PCOS functional and classification markers were merged and regarded as HA PCOS or NA PCOS markers.

### Discovering the characteristic difference between HA and NA PCOS

Co-occurrence analysis was applied on HA PCOS and NA PCOS up-regulated with common metabolism terms appeared on PCOS patients (Fig. 4d). HA PCOS was more correlated with obesity, vitamin D deficiency, hyperandrogenism, hirsutism, depression, insomnia and cardiac diseases. However, NA PCOS was more correlated with inflammation, immunity and insulin resistance. Meanwhile, we were interested on the relationship with feminine cancer for HA and NA PCOS. Survival p value (Fig. 4e) and hazard ratio (Fig. 4f) were calculated for HA and NA PCOS on 4 types of feminine cancers using GEPIA database [35]. No substantial difference was found on HA and NA PCOS on feminine cancer risks.

### Causal network construction for HA/NA PCOS markers and female infertility

**Fig. 5:**
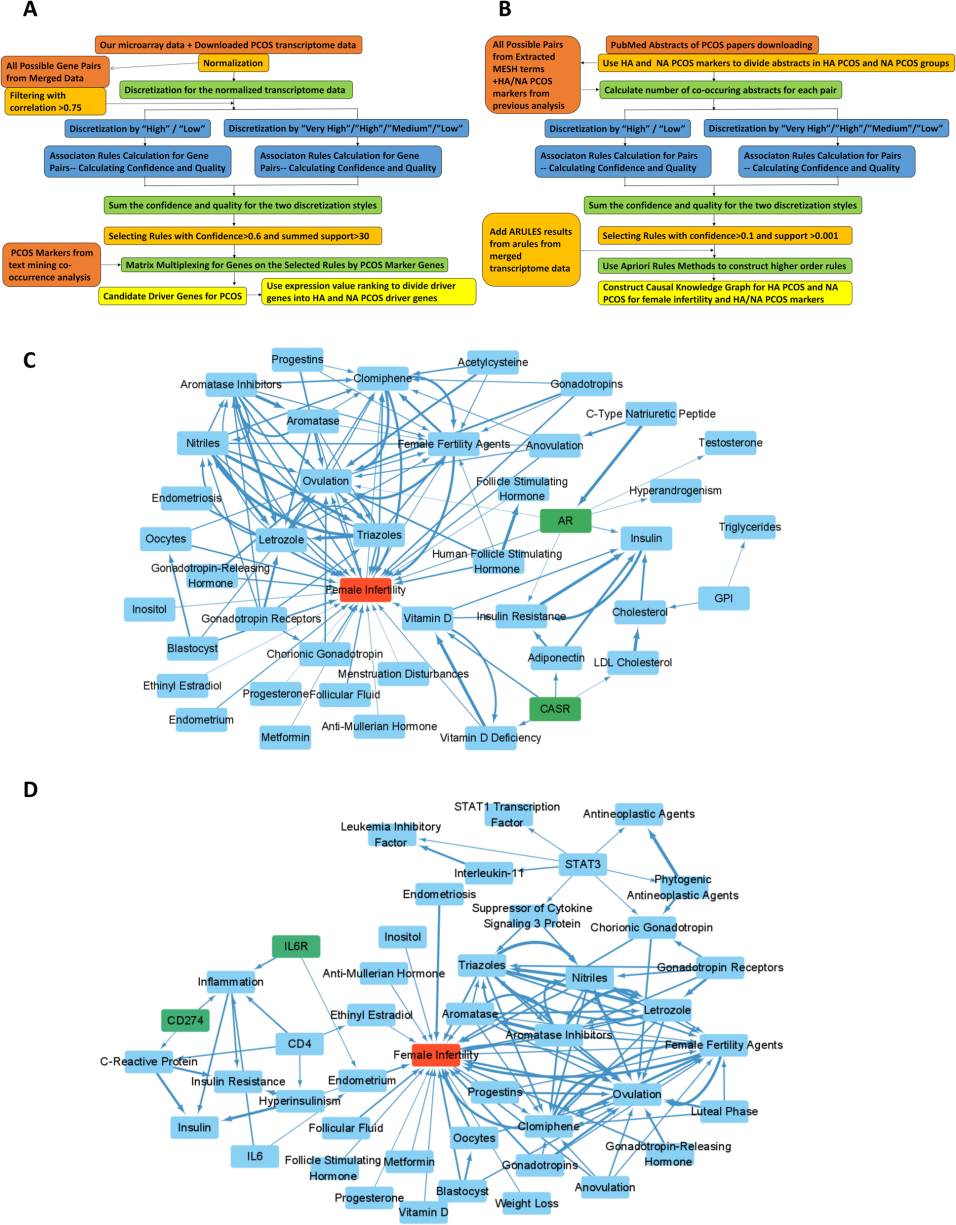
Constructing Knowledge Graph Using Association Rules Methods on HA and NA PCOS for Female Infertility. **A** Flowchart on the analysis process applying association rules algorithm on merged data of our microarray and downloaded PCOS datasets on HA and NA PCOS. **B** Flowchart on the analysis process applying association rules algorithm on downloaded PubMed abstracts on HA and NA PCOS. **C**, **D** Knowledge graph plotted by Cytoscape using causal relationship predictions by Apriori Rules Algorithm for HA PCOS (**C**) and NA PCOS (**D**) differentiated terms. The arrow implied causal relationship pointing from the cause side (upstream side) to the result side (downstream side). The HA and NA PCOS potential marker was labeled green. Female Infertility was labeled red. Width of lines implied confidence of the causal relationship

The subsequent question to be solved was how HA or NA PCOS markers resulted in female infertility. An effective tool to solve the problem was the Apriori Rules Algorithm [18, 19] which was specifically designed to calculate causal relationship between terms (refer to Methods). The causal relationship might be promoting or inhibiting relationship. The algorithm was applied on two different form of data: merged transcriptome data (Fig. 5a) and PubMed paper abstracts (Fig. 5b). Transcriptome data were using the merged transcriptome data from the final step of classification markers identification, and the causal relationship between each pair of genes were calculated. For abstract data, 9832 PubMed paper abstracts on PCOS from the last 12 years were downloaded. NA PCOS and HA PCOS up-regulated genes were used to classify the downloaded abstracts as NA PCOS papers and HA PCOS papers. NCBI MeSH words [45] with specific subheadings were applied as the measurement for the following Apriori Rules calculation. The number of MeSH terms co-occurred on the same abstract with each specific gene or MeSH term were recorded. Causal relationship was afterwards calculated based on the co-occurred number of MeSH terms. Apriori Rules method was then applied on calculated causal relationship to construct higher order rules with 3 elements for NA PCOS and HA PCOS. The term “Female infertility” was designed as the terminal for the causal cascade.

The 3 element rules ending as “Female infertility” were combined and merged with the rules from merged transcriptome data. Genes or terms with less connections were removed, and afterwards the the causal knowledge graph for HA PCOS (Fig. 5c) or NA PCOS (Fig. 5d) with female infertility was constructed. Mutual terms of HA and NA PCOS were removed from the knowledge graph (Fig. S5b). From the knowledge graph, we were capable of deriving the causal route for HA or NA PCOS markers and important MeSH terms which finally resulted in female infertility. The arrows on the network pointed from the cause to the effect (promote or suppress), or the sub-term to the term. Most of the causal relationships on the network were accordant with public knowledge and published papers. Unreported causal relationship might be prediction of the hidden mechanism for HA and NA PCOS. From the knowledge graph, we noticed pathogenesis for HA and NA PCOS was basically consistent including important process of ovulation, vitamin D, metabolic disorder and insulin resistance, etc., while however, pathogenesis of NA PCOS was more related to inflammation, and pathogenesis of HA PCOS was more related to androgen, hormone disorder and excessive adipose. Also from the knowledge graph, we noticed the important role for our newly discovered HA and NA PCOS functional markers IL6R, CD274 and CASR on female infertility.

### Drug interaction analysis predict specific drugs for HA and NA PCOS

**Fig. 6:**
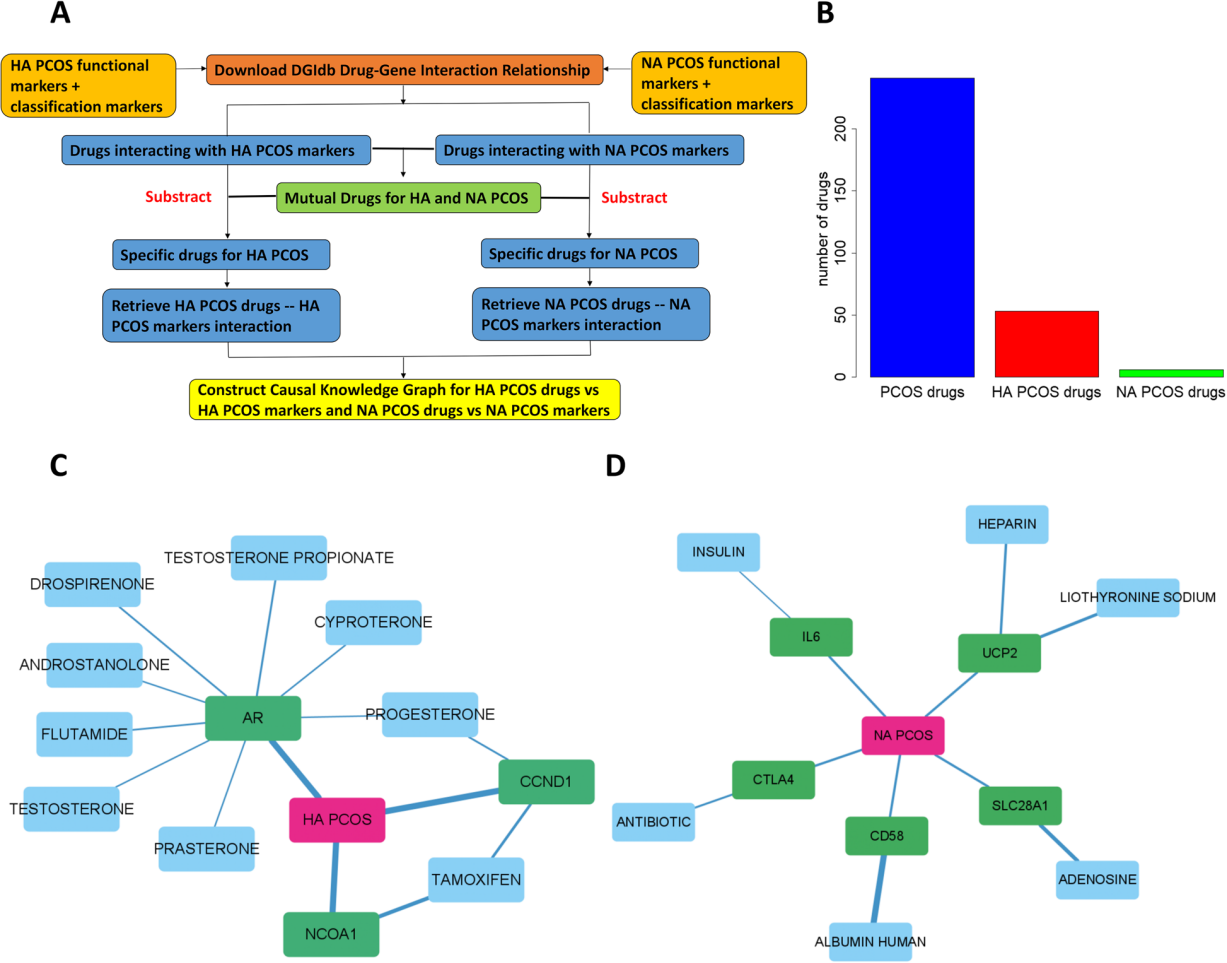
Applying Drug-Gene Interaction on Screening Interacted Drugs for HA and NA PCOS. **A** Flowchart on applying drug-gene interaction relationship on screening interacted drugs specifically for HA PCOS and NA PCOS. **B** Number of drugs for PCOS, drugs for HA PCOS and drugs for NA PCOS. **C**, **D** The network illustrating drug-gene interaction for interactive HA PCOS drugs vs HA PCOS markers (**C**) and for interactive NA PCOS drugs vs NA PCOS markers (**D**). HA/NA PCOS markers were labeled green, and drugs were labeled blue. Width of lines implied group interaction score between drug and interacting gene

The next clinical question to answer was the difference in drugs applied in HA and NA PCOS. We downloaded and applied the reported drug-gene interaction relationship from GRNdb [36] database on identifying the drugs specifically interacted with HA markers and NA markers. The flowchart of the analysis was shown (Fig. 6a). 23 HA PCOS specific drugs and 6 NA PCOS specific drugs were discovered (Fig. 6b). The co-occurrence counts with PCOS were illustrated (Fig. S5c,d). Further filtering removed drugs with less connections with marker genes. Then we constructed network based on the drug-gene interaction between selected drugs and HA/NA PCOS markers for HA and NA PCOS (Fig. 6c,d). The selected drugs were promoting or restraining the corresponding interacted markers. From the interaction network, we noticed androgen composed in most part, which was consistent with HA PCOS characteristics and probably promoting HA PCOS, while we also discovered flutamide and tamoxifen as specific drugs promoting or inhibiting HA PCOS. Within these drugs, tamoxifen had the potential to inhibit HA PCOS and promote ovulation and pregnancy [46]. For NA PCOS, we found human albumin, herapin, insulin, adenosine, liothyronine sodium and antibiotic might promote or inhibit NA PCOS. According to reported drug function, liothyronine sodium might promote NA PCOS [45], while antibiotic might potentially inhibit NA PCOS [47]. Human albumin might also inhibit NA PCOS by regulating immunity. Further drug experiments were required for detecting the potential drug targets suitable for HA or NA PCOS therapy.

## Discussion

Considering the vacancy on the studies on HA PCOS and NA PCOS, we applied microarray on granulosa cell samples for HA PCOS, NA PCOS and normal patients. Differential analysis identified specific genes for HA PCOS and NA PCOS. Further deeper functional analysis selected IL6R and CD274 as new NA PCOS functional markers, and meanwhile selected CASR as new HA PCOS functional marker. RT-qPCR and Western blotting validated that the expression pattern of IL6R, CD274 and CASR on mRNA and protein level were accordant with microarray data. Classification markers for HA PCOS, NA PCOS and normal groups were identified. Afterwards, combined methods of Apriori Rules and co-occurrence analysis were applied on downloaded PubMed PCOS abstracts. The causal network disclosed how HA PCOS or NA PCOS markers resulted in female infertility. Further drug-gene interaction analysis selected specific drugs promoting or inhibiting HA and NA PCOS. Our work was to our knowledge the first to apply microarray or sequencing technology on NA PCOS and HA PCOS mRNA samples, and among the first on applying transcriptome data on screening NA PCOS and HA PCOS markers and drugs, and thus our work was with considerable clinical significance. The methods applied in our studies were original methods and capable of detecting causal or interactive relationship between different types of datasets including transcriptome data, drug data and text mining data. The results also showed that CASR might play important role in HA PCOS progression, and IL6R and CD274 might dramatically engage in NA PCOS progression. Moreover, the results showed that diagnosis and treatment for HA PCOS and NA PCOS were different. For diagnosis, inflammatory indicators were feasible in NA PCOS diagnosis, and meanwhile glucose metabolism indicators or adipose indicators were feasible in HA PCOS diagnosis. For treatment,anti-inflammation drugs might be applied in NA PCOS treatment, and meanwhile anti-adipose or anti-androgen drugs might be applied in HA PCOS treatment.

Meanwhile, HA PCOS and NA PCOS markers have not been reported. Our work successfully identified NA PCOS markers IL6R and CD274, as well as HA PCOS markers CASR. Works from other researchers discussed the relationship of some of these markers including IL6R and CASR in PCOS [48, 49], but these work failed to describe the mechanism for the relation and also barely explain why they were expressed differently in NA and HA PCOS. Within these potential markers, CD274 (also named PDL1) is a special gene both identified by our method as NA PCOS specific gene and reported to be related to immunity and inflammation [39–41], and might indicate important implications between NA PCOS and tumor. Similarly, CASR was reported to be highly related to PCOS [42–44] and also correlated with adipose [50]. Our study might further imply internal relationship among HA PCOS, adipose and insulin. Thus identifying and experimentally validating potential markers for NA and HA PCOS might dramatically promote the understanding of PCOS and assist disclosing the mechanism that resulted in distinct syndrome of NA PCOS and HA PCOS.

Previous studies showed the similarity in syndrome for HA and NA PCOS patients including high levels of body mass index, insulin, total cholesterol and etc. [51]. Other works were concentrated on the difference for HA and NA PCOS and stated that HA PCOS patients possessed higher irisin level, adipocytokines and maternal complications [11, 52–54]. Increasing number of evidence showed the high correlation between PCOS and inflammation [55–58], and also the correlation between PCOS and vitamin D has been reported [59, 60], while however these studies were not distinguishing PCOS between subtypes of NA PCOS and HA PCOS. The main difference between NA PCOS and HA PCOS was still dilemma. Our causal knowledge graph showed that the causing pathogenesis for female infertility for HA PCOS and NA PCOS was mostly similar. Insulin / insulin resistance, aromatase / aromatase inhibitors, gonadotropin, inositol and even vitamin D were appearing on both HA PCOS and NA PCOS causes (Fig. 5c,d). This implied that the mechanism on how HA PCOS and NA PCOS caused female infertility might be similar. The specific cause for female infertility on HA PCOS included hyperandrogenism, cholesterol and adiponectin, while specific cause for NA PCOS included immunity terms and hyperinsulinism, indicating that HA PCOS was more correlated to metabolism disorders while however NA PCOS was more correlated to immunity disorders.

Our work is primarily based on bioinformatical and data mining technology, and thus our work is a prediction of the pattern, potential markers and specific drugs for NA PCOS and HA PCOS. The reliability of our conclusion is based on the fact that high percentage of our prediction was accordant with public or reported knowledge. It would be interesting for us to further illuminate the deeper mechanism underlying the function of IL6R, CD274 and CASR, and also HA/NA PCOS classification markers on the formation of PCOS and female infertility. Meanwhile, the specific HA and NA PCOS drugs on HA and NA PCOS were requiring further drug testing and might be of substantial clinical value.

### Supplementary Information

**Additional file 1: Table 1.** Clinical information for the patients of the collected granulosa cell samples.

**Additional file 2: Table S2.** Primers used for RT-PCR experiment.

**Additional file 3: Figure S1.** Quality assessment of our microarray analysis results. (A) Box Whisker Plot showing the average expression value of all samples. (B) Matrix plot illustrating the correlation of either of the two samples for all samples. (C) 2D PCA figure in showing the distribution of all samples. NA samples were labeled in color green, HA samples were labeled in color blue, and normal tissue were labeled in red. (D) Sample cluster analysis results for all samples. Distance between two samples showed the level of similarity within them. Samples with label ended with letter ‘d’ were NA samples, and also letter ‘h’ for HA samples, and letter ‘c’ for normal tissues.

**Additional file 4: Figure S2.** Supplemental Figures for PCOS Data Acquisition and Gene Ontology analysis results for HA PCOS specific genes. (A) Boxblot showing the PubMed co-occurrence analysis with the four subtypes of ovarian cells as keyword and the keyword “PCOS”, “HA PCOS” and “NA PCOS”. (B) Pearson’s correlation map showing correlation of all samples for each gene. (C) Pie plot illustrating the percentage of glucose metabolism terms within HA PCOS specific genes gene ontology terms. (D) STRING multi-protein network for the four candidate marker genes. The intermediate proteins were generated using the “more” function on STRING once. (E) PubMed co-occurrence results for SOX4 with PCOS, NA PCOS, HA PCOS and androgen receptor respectfully.

**Additional file 5: Figure S3.** Supplemental Figures for Gene ontology analysis results for NA PCOS specific genes. (A) Barplot showing the all DAVID biological process analysis terms within the NA PCOS specific genes. Immunity terms and immunity related terms were labeled red and green, and other terms were labeled as blue (Figure S2b-f were using the identical color labeling as Figure S2a). (B) Pie plot illustrating the percentage of immunity terms, immunity-related terms and other terms within all DAVID biological process analysis terms. (C) Barplot showing the top 20 DAVID KEGG pathways within the NA PCOS specific genes. (D) Pie plot illustrating the percentage of immunity pathways, immunity-related pathways and other pathways within the top 20 DAVID KEGG pathway analysis results. (E) Barplot showing all DAVID KEGG pathways within the NA PCOS specific genes. (F) Pie plot illustrating the percentage of immunity pathways, immunity-related pathways and other pathways within all DAVID KEGG pathway analysis results.

**Additional file 6: Figure S4.** Deep Analysis on NA PCOS GO terms to further identify potential NA PCOS markers. (A) Barplot illustrating the NA PCOS specific genes (x-axis) and the corresponding number of immunity GO terms which each gene belonged (y-axis). The genes existed on at least 5 immunity GO terms were plotted. (B) STRING network for the NA PCOS specific genes on at least 5 immunity GO terms. Connection with the type experimental validation and database were reserved. The genes with no connection with other genes were removed. (C) Barplot illustrating the NA PCOS specific genes (x-axis) and the corresponding number of top 20 immunity GO terms which each gene belonged (y-axis). The genes existed on at least 2 immunity GO terms were plotted. (D) STRING network for the NA PCOS specific genes on at least 2 of top 20 immunity GO terms. Connection with the type experimental validation and database were reserved. The genes with no connection with other genes were removed.

**Additional file 7: Figure S5.** Further analysis on merged PCOS data to extract deeper information. (A) Heatmap showing the expression pattern of HA PCOS, NA PCOS and NM groups after merging with downloaded PCOS datasets for each sample on x axis and each gene on y-axis. (B) Knowledge graph plotted by Cytoscape using causal relationship predictions by Apriori Rules Algorithm for HA PCOS and NA PCOS mutual terms. The arrow implied causal relationship pointing from the cause side (upstream side) to the result side (downstream side). The HA and NA PCOS potential marker was labeled green. Female Infertility was labeled red. Width of lines implied confidence of the causal relationship. (C) Co-occurrence counts (log) on PCOS for specific drugs on HA PCOS. (D) Co-occurrence counts (log) from co-occurrence on PCOS for specific drugs on NA PCOS.

## Data Availability

All data generated or analyzed during this study are included in this published article or in the data repositories listed in References. Microarray data were uploaded on NCBI Gene Expression Omnibus with Accession Number GSE137684.
