# Supplementary material for: Knowledge graph construction based on granulosa cells transcriptome from polycystic ovary syndrome with normoandrogen and hyperandrogen
Source: J Ovarian Res. 2024 Feb 12;17:38. doi: 10.1186/s13048-024-01361-z (PMC10860235; doi:10.1186/s13048-024-01361-z)
Supplement: Supplementary file 1 — Additional file 1: Table 1. Clinical information for the patients of the collected granulosa cell samples. [file 13048_2024_1361_MOESM1_ESM.docx]

**Table 1: Clinical Information for the Patients of the Collected Granulosa Cell Samples**

| *Clinical parameter* | *Control(N=4)* | *PCOS* | |
| --- | --- | --- | --- |
|  |  | *High T(N=4)* | *Low T(N=4)* |
| *Age* | 29.75±1.25 | 27.75±2.016 | 32±1.225 |
| *LH (IU/L)*  *FSH (IU/L)*  *LH/FSH* | 4.201±0.933  7.343±0.418  0.449±0.031 | 7.473±1.757^a^  7.055±0.6066  1.033±0.797 ^a^ | 7.054±0.134 ^a^  6.975±0.4336  1.028±0.811 ^a^ |
| *E2 (pmol/L)* | 75.14±39.41 | 163.3±39.54 | 57.89±24.77 |
| *P (nmol/L)* | 0.825±0.2513 | 1.47±0.7073 | 0.3325±0.049 |
| *PRL(ng/ml)* | 12.03±3.321 | 10.07±2.524 | 14.59±3.215 |
| *lh(IU/L)* | 5.663±1.197 | 10.38±3.774 | 6.803±1.458 |
| *T(ng/ml)* | 0.215±0.0883 | 0.5375±0.0792* | 0.065±0.023 |
| *FBG* | 5.643±0.2447 | 5.455±0.1869 | 5.503±0.183 |
| *Weight* | 66.38±6.074 | 56.13±3.112 | 64.25±3.179 |

Student *t-test, *p<0.05；a p<0.05 vs* control.
