# Supplementary material for: Knowledge graph construction based on granulosa cells transcriptome from polycystic ovary syndrome with normoandrogen and hyperandrogen
Source: J Ovarian Res. 2024 Feb 12;17:38. doi: 10.1186/s13048-024-01361-z (PMC10860235; doi:10.1186/s13048-024-01361-z)
Supplement: Supplementary file 2 — Additional file 2: Table S2. Primers used for RT-PCR experiment. [file 13048_2024_1361_MOESM2_ESM.docx]

**Table S2 Primers used for RT-PCR experiment**

| Gene | 5'primer | 3'primer |
| --- | --- | --- |
| CASR | AACGCTTGACCTGAGTCTTG | ATGGTTCTGCCGTCTCTCCA |
| IL-6R | TTGCCATTGTTCTGAGGTT | GTAGTCTGTATTGCTGATGTC |
| CD274 | CGTGACAAGAGGAAGGAAT | AGGATGAGCAATGGATGATT |
| BMP2 | CGAAATTCCCCGTGACCAGA | TGTTTCTCCTCCAAGTGGGC |
| GDF6 | GAGGGTGTATGCGACTTCCC | GAGTCAATTTGGTGGGCACG |
| CYP2G1P | CCTTCACTCCAAGCTCAGGG | CCGCTTGGTCTATCAAGGCT |
| CYP3A7 | TGCATTGGCATGAGGTTTGC | CAGGCTCCACTTACGGTCTC |
| DMRTC1 | CTCCAGTGACCTTGGAGCAG | GAGACTTTGGGGACCTGTGG |
| FGFR1 | GCCCAGACAACCTGCCTTAT | CACGTATACTCCCCTGCGTC |
| FSHR | GAGGCCTTCCAGAACCTTCC | ACTTTCAAAGCTCAGCCCCA |
| CYP2B6 | TTTGCCAATGGAAACCGCTG | GTTGGCGGTAATGGACTGGA |
| HSD17B3 | ATTTCCTGAACGCACCGGAT | GGCCAAGGAAACAGGGCTAT |
| IL27 | CAGGCGACCTTGGCTGG | GCTGACTGTGAACTCCCTCC |
| SOX4 | GACCTGAACCCCAGCTCAAA | AGCCGGGCTCGAAGTTAAAA |
| SOX5 | ACCACCCAAAAGCAAGGAAA | TGGGGTTCATTGCTACCACG |
| TGFB2 | CCCTTCTTCCCCTCCGAAAC | CAAGGTACCCACAGAGCACC |
