# Supplementary figures and images for: Knowledge graph construction based on granulosa cells transcriptome from polycystic ovary syndrome with normoandrogen and hyperandrogen
Source: J Ovarian Res. 2024 Feb 12;17:38. doi: 10.1186/s13048-024-01361-z (PMC10860235; doi:10.1186/s13048-024-01361-z)

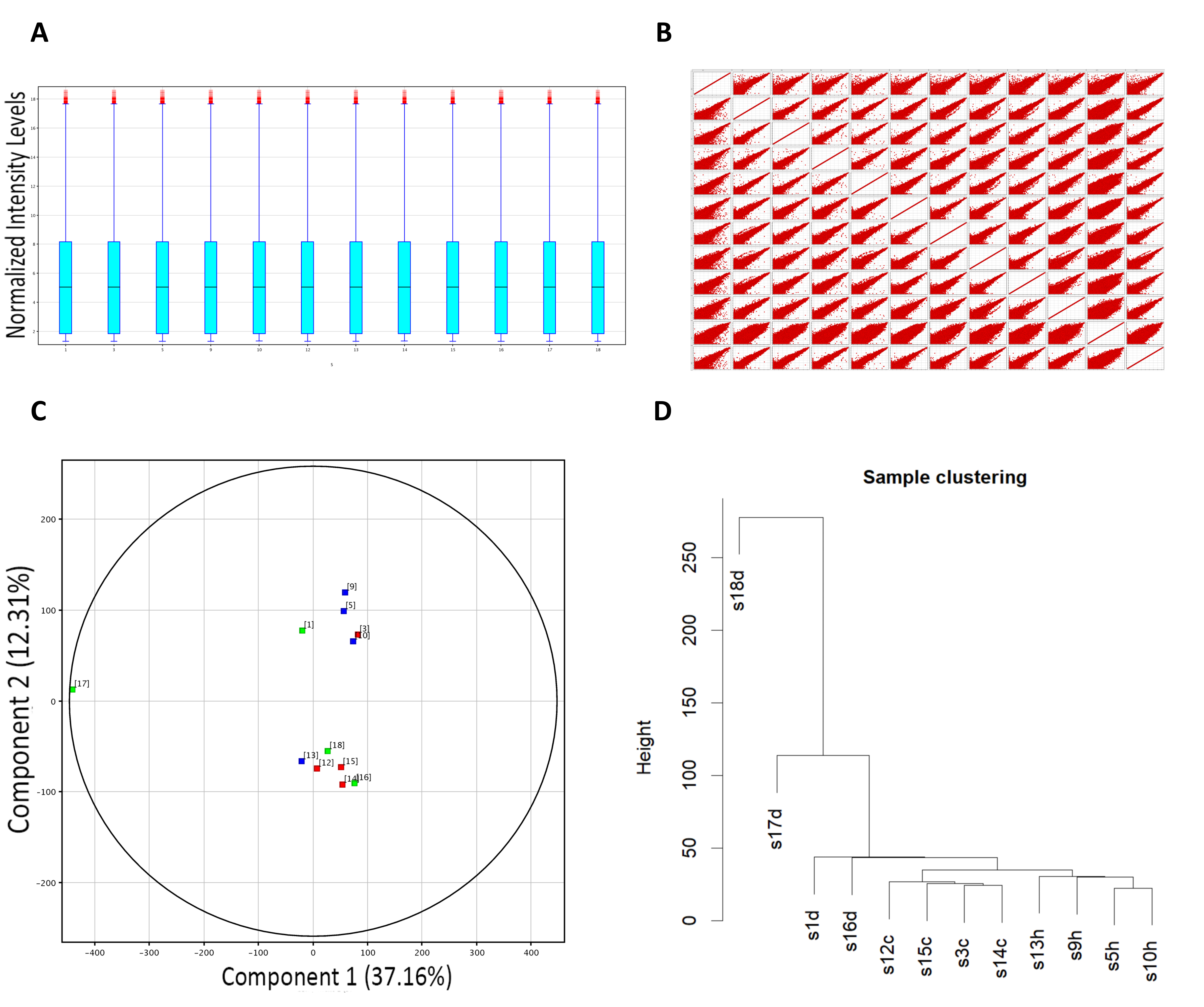

Supplement: Supplementary file 3 — Additional file 3: Figure S1. Quality assessment of our microarray analysis results. (A) Box Whisker Plot showing the average expression value of all samples. (B) Matrix plot illustrating the correlation of either of the two samples for all samples. (C) 2D PCA figure in showing the distribution of all samples. NA samples were labeled in color green, HA samples were labeled in color blue, and normal tissue were labeled in red. (D) Sample cluster analysis results for all samples. Distance between two samples showed the level of similarity within them. Samples with label ended with letter ‘d’ were NA samples, and also letter ‘h’ for HA samples, and letter ‘c’ for normal tissues. [file 13048_2024_1361_MOESM3_ESM.tif]

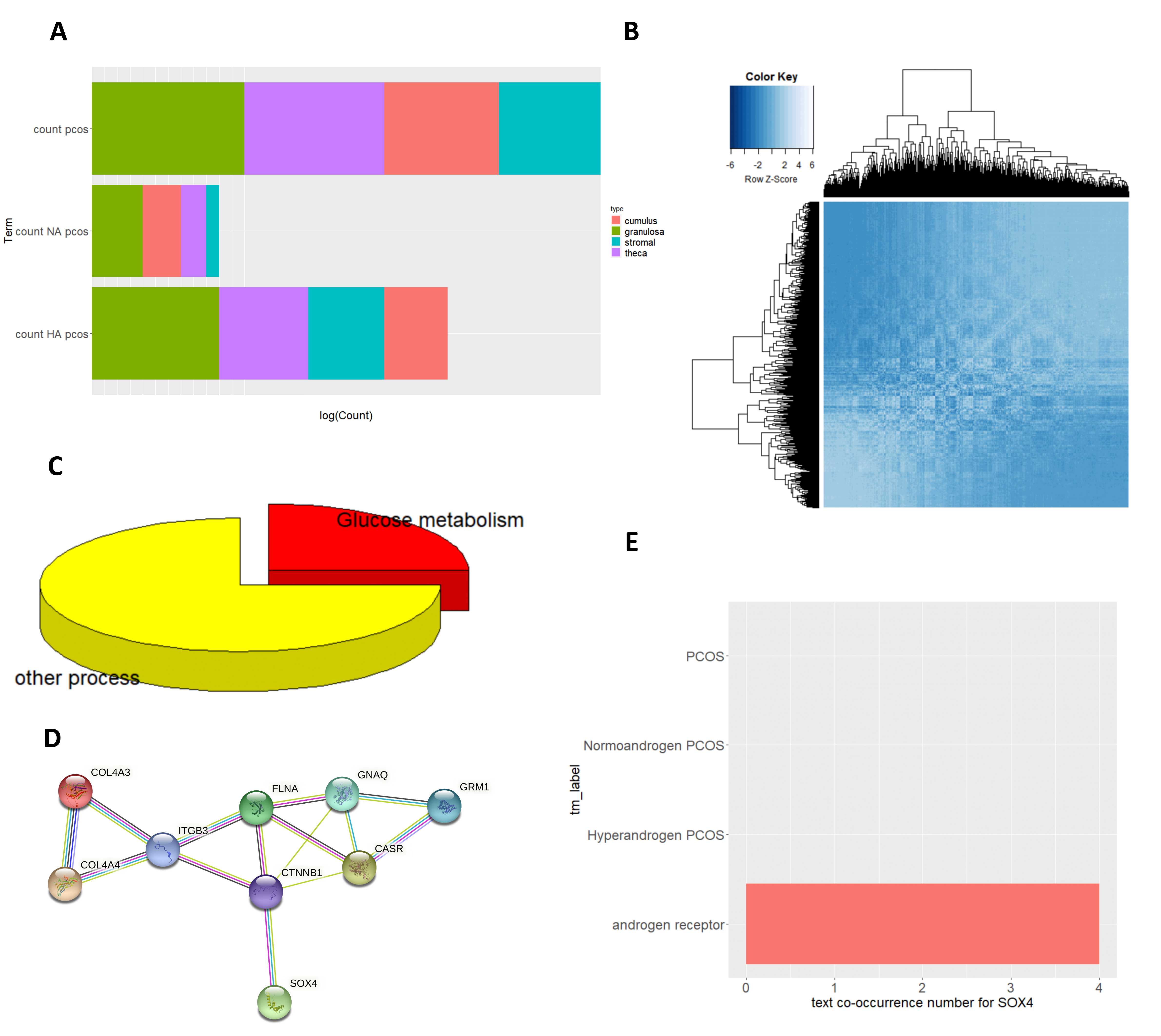

Supplement: Supplementary file 4 — Additional file 4: Figure S2. Supplemental Figures for PCOS Data Acquisition and Gene Ontology analysis results for HA PCOS specific genes. (A) Boxblot showing the PubMed co-occurrence analysis with the four subtypes of ovarian cells as keyword and the keyword “PCOS”, “HA PCOS” and “NA PCOS”. (B) Pearson’s correlation map showing correlation of all samples for each gene. (C) Pie plot illustrating the percentage of glucose metabolism terms within HA PCOS specific genes gene ontology terms. (D) STRING multi-protein network for the four candidate marker genes. The intermediate proteins were generated using the “more” function on STRING once. (E) PubMed co-occurrence results for SOX4 with PCOS, NA PCOS, HA PCOS and androgen receptor respectfully. [file 13048_2024_1361_MOESM4_ESM.tif]

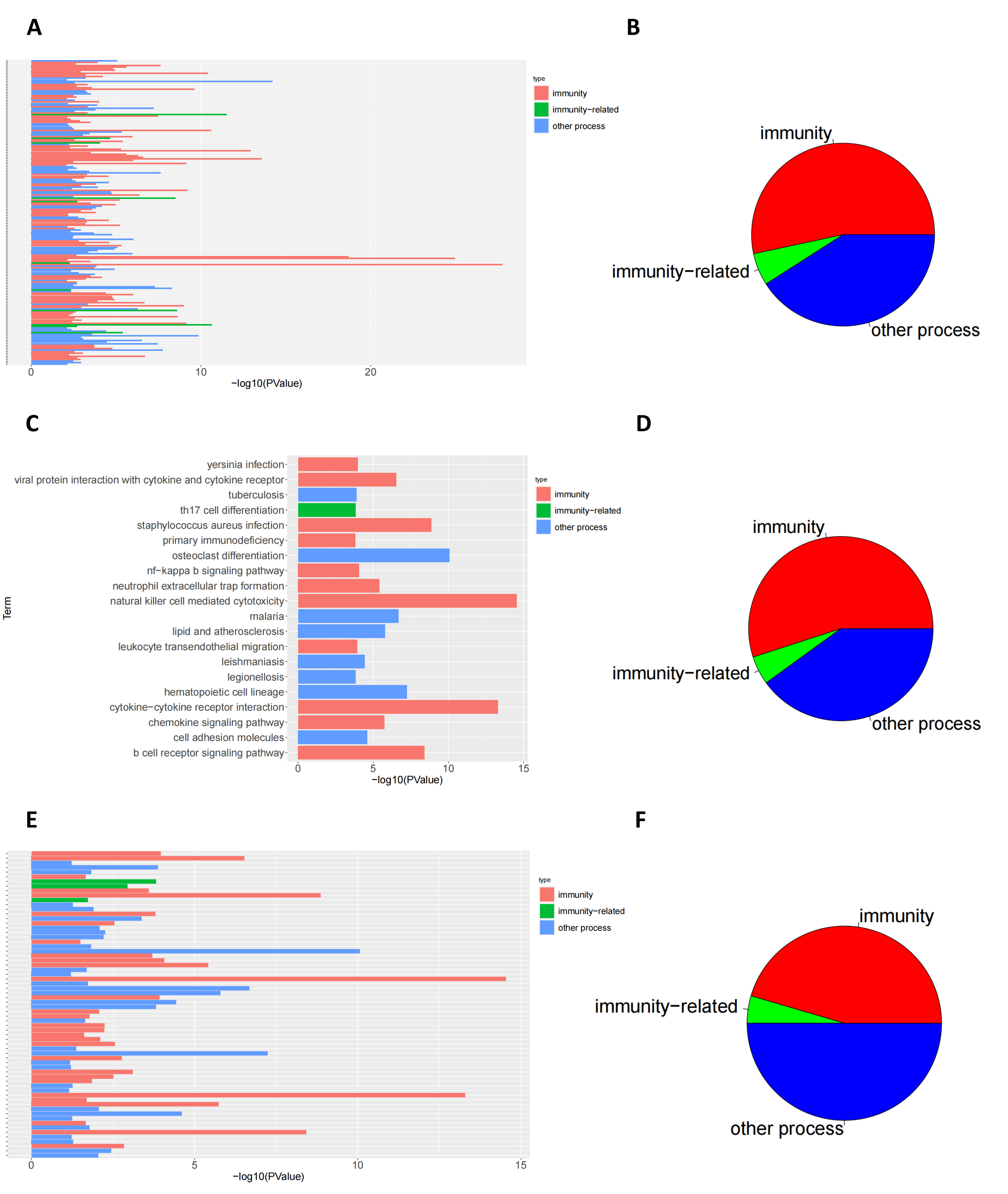

Supplement: Supplementary file 5 — Additional file 5: Figure S3. Supplemental Figures for Gene ontology analysis results for NA PCOS specific genes. (A) Barplot showing the all DAVID biological process analysis terms within the NA PCOS specific genes. Immunity terms and immunity related terms were labeled red and green, and other terms were labeled as blue (Figure S2b-f were using the identical color labeling as Figure S2a). (B) Pie plot illustrating the percentage of immunity terms, immunity-related terms and other terms within all DAVID biological process analysis terms. (C) Barplot showing the top 20 DAVID KEGG pathways within the NA PCOS specific genes. (D) Pie plot illustrating the percentage of immunity pathways, immunity-related pathways and other pathways within the top 20 DAVID KEGG pathway analysis results. (E) Barplot showing all DAVID KEGG pathways within the NA PCOS specific genes. (F) Pie plot illustrating the percentage of immunity pathways, immunity-related pathways and other pathways within all DAVID KEGG pathway analysis results. [file 13048_2024_1361_MOESM5_ESM.tif]

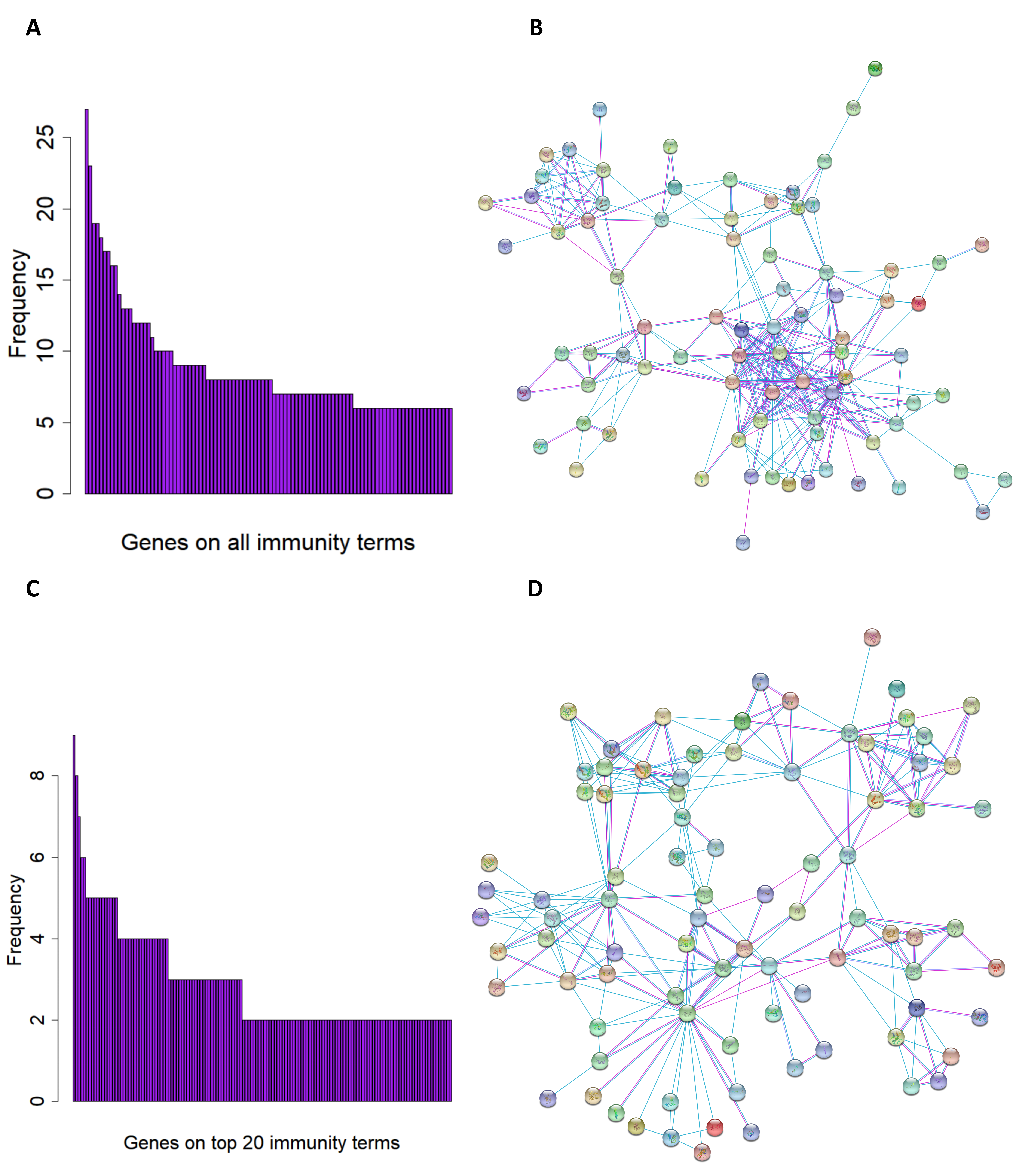

Supplement: Supplementary file 6 — Additional file 6: Figure S4. Deep Analysis on NA PCOS GO terms to further identify potential NA PCOS markers. (A) Barplot illustrating the NA PCOS specific genes (x-axis) and the corresponding number of immunity GO terms which each gene belonged (y-axis). The genes existed on at least 5 immunity GO terms were plotted. (B) STRING network for the NA PCOS specific genes on at least 5 immunity GO terms. Connection with the type experimental validation and database were reserved. The genes with no connection with other genes were removed. (C) Barplot illustrating the NA PCOS specific genes (x-axis) and the corresponding number of top 20 immunity GO terms which each gene belonged (y-axis). The genes existed on at least 2 immunity GO terms were plotted. (D) STRING network for the NA PCOS specific genes on at least 2 of top 20 immunity GO terms. Connection with the type experimental validation and database were reserved. The genes with no connection with other genes were removed. [file 13048_2024_1361_MOESM6_ESM.tif]

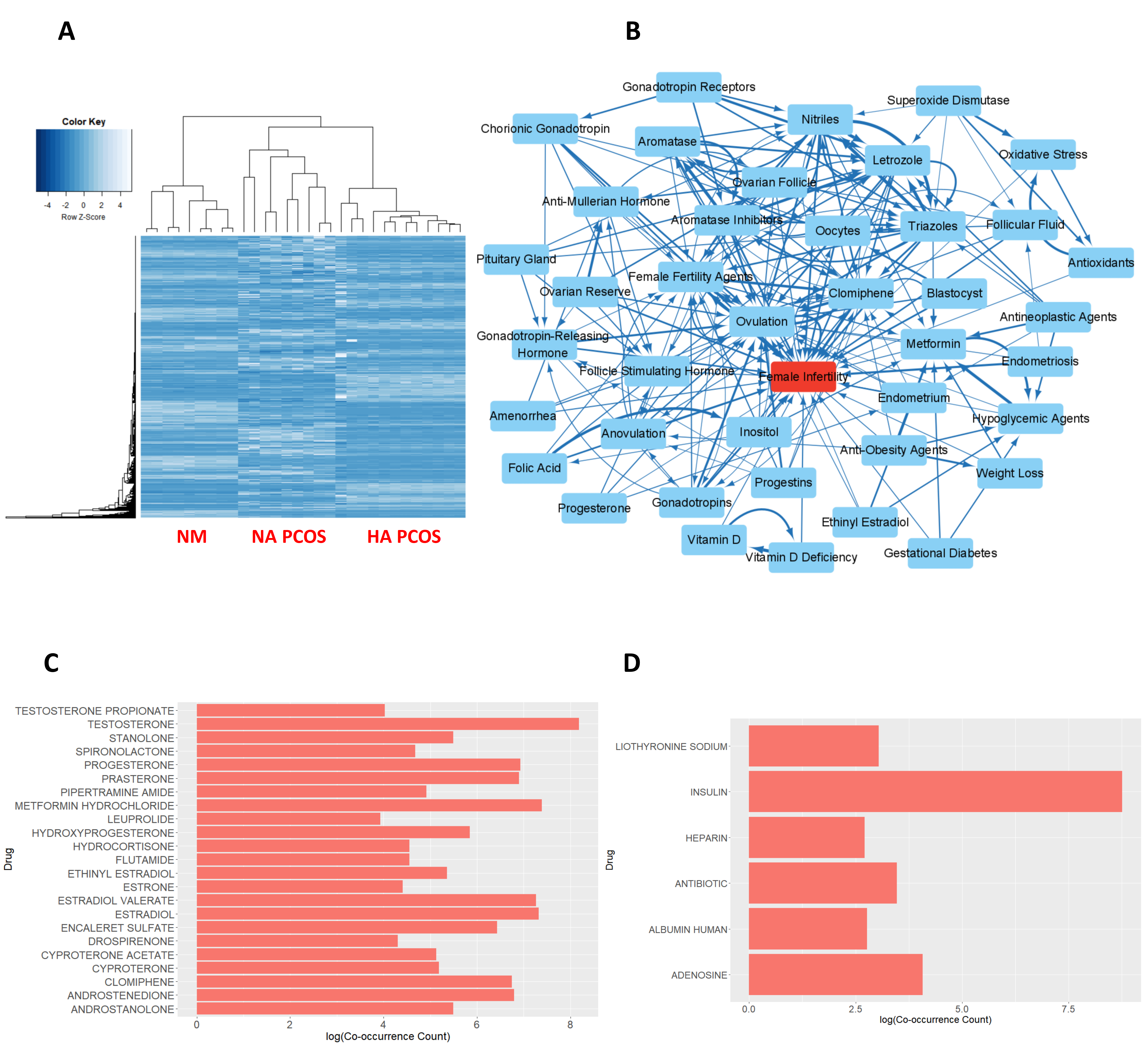

Supplement: Supplementary file 7 — Additional file 7: Figure S5. Further analysis on merged PCOS data to extract deeper information. (A) Heatmap showing the expression pattern of HA PCOS, NA PCOS and NM groups after merging with downloaded PCOS datasets for each sample on x axis and each gene on y-axis. (B) Knowledge graph plotted by Cytoscape using causal relationship predictions by Apriori Rules Algorithm for HA PCOS and NA PCOS mutual terms. The arrow implied causal relationship pointing from the cause side (upstream side) to the result side (downstream side). The HA and NA PCOS potential marker was labeled green. Female Infertility was labeled red. Width of lines implied confidence of the causal relationship. (C) Co-occurrence counts (log) on PCOS for specific drugs on HA PCOS. (D) Co-occurrence counts (log) from co-occurrence on PCOS for specific drugs on NA PCOS. [file 13048_2024_1361_MOESM7_ESM.tif]
